# Knowledge and Beliefs about Dengue Transmission and Their Relationship with Prevention Practices in Hermosillo, Sonora

**DOI:** 10.3389/fpubh.2015.00142

**Published:** 2015-06-03

**Authors:** Carmen Arellano, Lucía Castro, Rolando E. Díaz-Caravantes, Kacey C. Ernst, Mary Hayden, Pablo Reyes-Castro

**Affiliations:** ^1^Centro de Estudios en Salud y Sociedad, El Colegio de Sonora, Hermosillo, Mexico; ^2^College of Public Health, University of Arizona, Tucson, AZ, USA; ^3^National Center for Atmospheric Research, Boulder, CO, USA

**Keywords:** dengue, transmission, focus groups, U.S.-Mexico border, knowledge, attitudes

## Abstract

**Background:**

Dengue is an emerging threat in the U.S.-Mexico border region. Transmission has regularly occurred in Sonora, MX since 1982 but it was not until 2014 that cities directly on the Arizona-Sonora border had local transmission. One of the closest urban areas to have regular seasonal transmission is Hermosillo, SN, MX. Developing a better understanding of the knowledge and perceptions of dengue in close geographic proximity to the border can identify areas to target for prevention and control measures.

**Methods:**

We conducted focus groups in six neighborhoods in Hermosillo, SN, MX; three with high-dengue transmission and three with lower transmission. Awareness of dengue and experience with dengue was common.

**Results:**

In all focus groups, discussants reported knowing someone personally who had past dengue infection. We further identified several key ways that the perceptions of dengue transmission could influence the effectiveness of dengue control campaigns. First, there was confusion about how dengue is transmitted. While people associated dengue with mosquitoes, multiple modes of transmission were perceived including direct person-to-person transmission. In one focus group, discussants indicated a stigma surrounding dengue infection. The necessity to maintain cleanliness in their households was identified as a primary strategy to fight dengue; however, participants also noted the limited impact and their actions may have on transmission if there is lack of community support or governmental infrastructure to control neighboring and public spaces.

**Conclusion:**

As dengue risk increases in the border region, more efforts should be made to clearly convey the single mode of transmission of dengue to avoid the development of stigma. More coordinated efforts should be made to not only control but also prevent dengue.

## Introduction

The dengue virus, transmitted through the bite of the *Aedes aegypti* mosquito, has spread widely across the globe over the past several decades. An estimated 390 million people are infected every year (1). In Americas, dengue transmission has expanded rapidly in both geographic distribution and incidence (1). Dengue manifests with high fever, skin rash, headache, retro-orbital pain, and in severe cases it can lead to hemorrhaging and death. In Mexico, dengue is endemic with a seasonal peak following the monsoon rains. Transmission intensity generally declines moving north from the hot and humid southern region of Mexico (2) to the northern, more arid region of Mexico including the state of Sonora in the U.S. – Mexico border. Dengue has been reported in Sonora since 1982 (3). Most years Sonora has only a modest number of cases; however, in 2010, over 4,000 laboratory-confirmed cases were reported with 7 deaths (4, 5). In 2014, a similar outbreak occurred with several thousand laboratory-confirmed cases and hundreds of dengue hemorrhagic manifestations (6). Most research on dengue has focused on tropical areas where the transmission rate is high and the communities have extensive personal experience with the disease. Few studies have examined community awareness of dengue in arid regions, such as Hermosillo, SN, MX, which is experiencing increasing transmission.

The objectives of the study were (1) to assess knowledge and beliefs about dengue transmission and (2) to gain insight into dengue prevention practices in Hermosillo, SN, MX. Hermosillo is also the closest city to the Arizona-Sonora border with regular seasonal transmission. Six focus groups were conducted to obtain information from neighborhoods with diverse socio-economic status and different levels of dengue transmission. We present information on the knowledge and beliefs about preventing dengue in the household, accessing healthcare when ill, how dengue is transmitted, risk factors for dengue, and the role climate change has on transmission. The study is a binational collaboration between the University of Arizona and El Colegio de Sonora.

## Theoretical Background

Wellbeing/illness/care (w/i/c) is not just a medical but also a cultural process. According to Menéndez (7), the w/i/c processes are “social facts on which social groups need to build actions, techniques, and ideologies… collective social meanings.” Based on this premise, we recognize that collective knowledge is socially shared.

This social representations theory provides a theoretical framework to analyze the process of w/i/c surrounding dengue. The concept of social representation is a contribution of Serge Moscovici to the field of social psychology (8). It was first used in 1960s to refer to “an organized body of knowledge and a psychological activity through which people make the social and physical reality understandable,” in other words, group interactions that make the unfamiliar familiar (9). This definition allows us to analyze the collective social thinking as a system of values, practices, and ideas that gives order and communication among people from a social group. This theory collates other concepts such as attitude, belief, image, stereotype, and perception, related to cognitive processes that create an “elaborated and shared social knowledge” (9).

According to Moscovici, three elements of social representation can be analyzed: (1) *information*, which is the quantity and quality of shared knowledge among social group members that drives their interpretation of reality; (2) the *field of representation*, which refers to the order and organization of the content of representation that integrates a social model and image of the representation; (3) *attitude* or behavioral elements related to the agreement or disagreement with the object of the social representation, in this case, dengue.

Based on these three elements of social representation, in this study, we identified knowledge and beliefs of participants in the six focus group discussions (FGDs) about the mode of dengue transmission and their relation to themes about prevention practices.

## Methodology

Focus groups were carried out to identify current discussion, beliefs, values, and attitudes about dengue in neighborhood groups in Hermosillo, SN, MX (10). This study was reviewed by the University of Arizona Human Subjects committee and was deemed exempt. El Colegio de Sonora deferred IRB oversight to the University of Arizona. All focus group participants were read a disclosure statement but written consent was not obtained to minimize any connection between participants and responses. Participation was voluntary and did not include compensation. The use of qualitative methods can provide information on how the perception of dengue is constructed in these communities. These findings can inform public health interventions surrounding prevention and treatment and take into consideration the cultural significance and interpretation of concepts of well-being and illness in diverse environmental settings with differing levels of dengue transmission. This dynamic can be applied to similar communities in the border region that have yet to experience dengue transmission.

### Study methods

We conducted 6 FGDs; ranging from 6 to 10 participants. Common topics were explored among all focus groups to determine distinct perspectives and to identify areas of agreement and disagreement among communities (11). A semi-structured guide was created *a priori* to direct the discussion. Topics covered four primary areas (1) perception of risk of dengue, (2) awareness and knowledge of the disease, (3) factors perceived as increasing risk of disease, and (4) prevention and control strategies in which community members were engaged.

### Sampling and recruitment of participants

**Figure 1:**
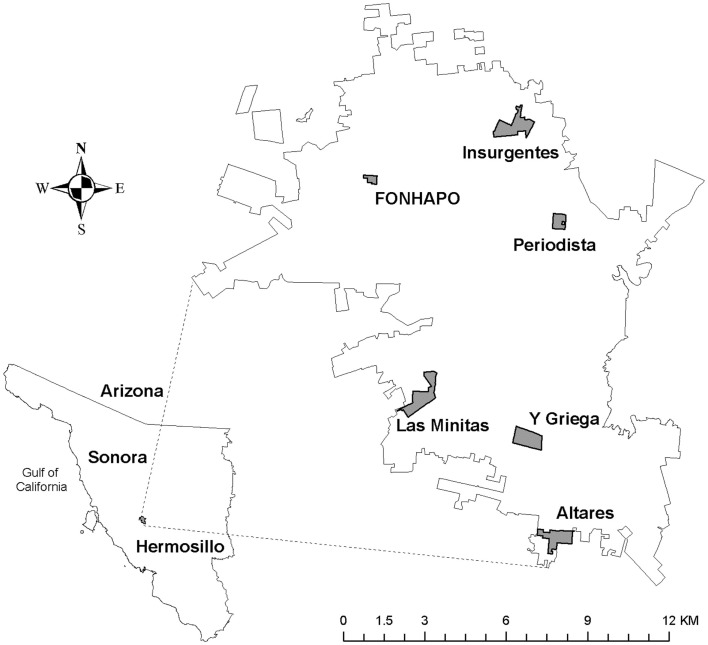
**Study sites, six neighborhoods identified in Hermosillo, SN, MX for carrying out the focus group discussions**.

Purposive, stratified sampling of neighborhoods was conducted to identify neighborhoods with different levels of socio-economic status, education, and dengue incidence. The selected neighborhoods with high incidence (HI) were Insurgentes, Minitas, and Periodista; while Fonhapo, Y Griega, and Altares were part of the low incidence (LI)^1^ group (see Table 1; Figure 1).

**Table 1:** **Study area: six neighborhoods of Hermosillo, SN, MX**.

Dengue incidence	Neighborhoods
High	Insurgentes
	Minitas
	Periodista
Low	Altares
	Fonhapo
	Y Griega

Initial support was sought from female community leaders and community center personnel (the FGDs were conducted in community centers). Walkthroughs in targeted neighborhoods were undertaken to invite people to the FGDs. Verbal informed consent of all of the participants was requested during which the voluntary nature of participation and the confidentiality of their information was reiterated. Permission to record the discussions was requested and they were subsequently transcribed and coded using the NVivo version 7 software.

### Site description/socio-demographic context

Socio-demographic conditions of the six study areas are shown in Table 2. The neighborhoods with higher population density were Insurgentes (120 persons/hectare) and Las Minitas (98 persons/hectare) both categorized as high-dengue incidence areas. However, there were fewer differences among neighborhoods in terms of average persons per house, which ranged from 3.3 (Periodista) to 4.0 (Insurgentes).

**Table 2:** **Socio-demographic characteristics of the six study areas by level of dengue incidence**.

Socio-demographic characteristics	High incidence			Low incidence		
	Insurgentes	Periodista	Las minitas	Fonhapo	Y griega	Altares
Total population	9,653	1,178	3,824	1,105	1,988	11,377
Number of blocks	101	26	67	18	39	104
Total area (ha)	80	40	40	30	80	240
Population density	120	27	98	43	25	47
Population with incomplete basic school ≥15 (%)	31.0	11.3	36.0	19.8	32.9	16.5
Population with no healthcare service (%)	28.8	14.3	23.2	17.8	24.7	16.8
Occupied particular houses	2,389	358	981	292	527	3,057
Average/persons houses	4.0	3.3	3.9	3.8	3.8	3.7
No piped water (%)	5.3	0.6	3.7	0	0.8	1.8
No toilet (%)	1.2	0.6	2.8	0.3	1.1	0.5
No drainage (%)	1.3	0.6	6.2	0.3	1.1	1.4
Dirt floor (%)	6.2	0.6	6.6	1	2.7	1.3
No electricity (%)	0.8	0.6	2.1	0	0.9	0.6

*Source: INEGI. 2010 Mexican Census*.

The neighborhoods with the highest proportions of the population with no basic education were Y Griega (32.9%), Insurgentes (31%), and Las Minitas (36%). Moreover, these same neighborhoods had the highest proportions of the population with no healthcare services (24.7, 28.8, and 23.2%, respectively).

In terms of housing conditions, Insurgentes had the highest proportion of houses with no piped water (5.3%). Las Minitas had the highest proportion of houses with dirt floors (6.6%), no drainage (6.2%), and no electricity (2.1%). Although the Periodista neighborhood had better socio-demographic conditions, it was categorized as a high-dengue incidence area in Hermosillo.

### Participant description

In this study, only self-reported heads of household were included since they are more likely to be the ones making healthcare decisions, including care practices and treatment seeking behaviors. In the 6 FGDs, a total of 68 people participated: 66 women and 2 men; the 2 men were from the Altares neighborhood. All the participants had at least one child. The average age of the participants was 38 years (the youngest was 22 and the oldest 74 years old).

Even though having suffered from dengue was not an inclusion criteria, seven of the participants had previously suffered from the disease; furthermore, in all six neighborhoods discussants reported knowing at least 1 person with a history of dengue diagnosis. Experiencing dengue directly or knowing someone who has had dengue may lead to a different perspective of the importance of dengue as compared to individuals who are only aware of the disease through information provided by health institutions or the media.

## Results

The main empirical findings were centered on two major themes: (1) beliefs about the way dengue is transmitted, and (2) dengue prevention strategies in household and community environments. We further distinguish between those strategies that are undertaken at a family and/or community level, and those implemented by governmental institutions.

### Beliefs about transmission

Participants’ beliefs about the way dengue is transmitted come from several sources: previous experiences with dengue itself, messages transmitted by the media, and preventive campaigns implemented by health institutions. In general, the participants were confused about the way dengue is transmitted. Despite identification of the mosquito as a source of transmission, alternative routes of transmission were also speculated, including person-to-person transmission. One participant commented, “*And they say it is contagious, right?*” (Y Griega-LI).

This idea of person-to-person transmission is played out in the collective imagination due to the participants’ recollection that often more than two people fell ill in the same household during simultaneous or consecutive periods. The direct experience with several sick people led participants to question if the transmission was due to a mosquito bite or by person-to-person contact, as is shown in the next testimony:

And when there are four people in the same place, are the four people infected by the same mosquito? or by others or how?… because my husband got sick (with dengue), later my mom got sick, later my aunt got sick, and so on four or five people living in the same house. It must be like the flu, I guess, right? (Altares-LI)

This statement suggests confusion regarding the transmission of dengue. It is unclear to participants if transmission is due to a mosquito bite or through direct contact with sick persons. They confuse the mechanism of dengue transmission with the transmission mechanism of other familiar viral diseases. This confusion is reinforced by knowing of multiple sick people within the same space-time. This idea is shared in the collective perceptions of participants from other neighborhoods and even among those with direct experience with dengue.

My father in law got sick and one or two days later I got sick too (Insurgentes-HI).

As mentioned above, disease perceptions go beyond the medical model and into a system of social representation integrated by meanings and beliefs around the w/i/c process. Despite having direct experience with the biological process of getting sick, some of these beliefs about the contagious nature of dengue are supported by the participants because key information is not presented in a clear and simple way. This has been noted in other disease systems as well (12). This perception may impact prevention/care practices further down the pathway of w/i/c. Confusion about the vector–host relationship was also expressed. The mosquito was identified as the vector; however, it was also implicated in transmitting non-vector borne diseases.

If you have the flu and a mosquito comes to bite you, and nothing happens to you but there is another weaker person. That’s when he gets sick.

And people said that our disease is transmitted and it is true… if a person has a disease, everything is transmitted to another person. That’s true. That’s why sick people should be isolated (Minitas-HI).

This belief is not only found in those with no experience with the dengue but even among those that have had direct or close experience with dengue. These data indicate the importance of doctor–patient communication. Beliefs about the disease could affect care, follow-up and future prevention practices. In two FGDs, participants mentioned the need to “isolate” the ill from other people to avoid spreading the disease:

Interviewer: “And how long did he remain sick (the son of one the informant)?”

Participant: “Eight days and for him not to have contact with the other kids, but he has to get out of the house, and it can’t be done.”

I: “What do you mean by keeping him isolated?”

P: “Yes, like keeping him isolated… because it is contagious.” (Altares-LI).

The idea of isolation was discussed in two ways, one way; as described earlier, was to avoid contact between those who are ill and other people to prevent direct transmission. Another way, points out the need to avoid contact between the sick person, the mosquito, and other people to whom the disease could be transmitted. This perception of the need for isolation was expressed as follows:

The ill need to be covered… be isolated from other people, covered with a mosquito net, or something like that, right? So that the mosquito doesn’t get back in and transmit it to the same people and have a period of 40 days of bed rest. (Insurgentes-HI)

This belief is mentioned throughout neighborhoods with both high- and low-dengue occurrence; the concept of isolation of ill people is part of social representations of dengue as a contagious disease. While dengue is not transmissible person-to-person, this concept is part of the local knowledge that people use to prevent disease and isolating the sick person may, in fact, reduce the number of mosquitoes that can be infected in a household. In one of the FGDs in a neighborhood with high occurrence, the participants contemplated blood contact as a way of transmitting the disease:

But if you get a cut and just happen to grab it, I imagine that then yeah [you could get dengue], because your blood mixes with the other blood. (Periodista-HI)

This idea about transmission through blood may be related to knowledge about how prevention of other viral diseases requires avoidance of contact with bodily fluids like blood and saliva. An example is HIV/AIDS, which is strongly stigmatized (13, 14). These beliefs about dengue transmission are exacerbated when a person dies from dengue; this prompts other measures to avoid transmission, as is shown in the following testimony:

They had to fumigate, throw away the bed of the kid, the clothes, they sterilized everything… do you know what people did? They distanced themselves, the people next door raised their wall, they protected themselves… maybe so that the same wouldn’t happen, right? Because one has to protect oneself, but it was so obvious that people distanced themselves. And actually only a few of us went to his burial. (Altares-LI)

There is a need to broaden the discussion about stigma and transmittable disease, because even though the participant in Altares condemns the act of distancing oneself, she herself makes reference to the need to protect oneself. What does protecting oneself against dengue imply? This confusion about the mode of transmission is likely what has led to this type of community reaction.

In all of the FGDs, the mosquito is identified as the carrier of the disease, but the ways through which dengue can be spread leads to confusion. In the Altares FGD, the participants discussed ideas and beliefs related to modes of transmission. At the end of the session, they mentioned this:

It is more about lack of information. For example, they told her that she could get infected, Others say that it is not contagious, that it should be [transmitted] by the bite. Then, what happens? Happens that we don’t know if it is contagious or not, or if the mosquito with the dengue virus is the only one able to transmit the disease (Altares-LI).

These ideas about alternative mechanisms of dengue transmission come from previous experiences and socially shared images about the disease. They are based on practical experiences dealing with other diseases and using that experience to interpret reality. Only in the Fonhapo FGD (neighborhood with low occurrence) was transmission through the mosquito bite referred to in a more definitive way:

I know that it is caused by a mosquito.

I believe that if there is a person with dengue and a mosquito bites them and that mosquito bites you, then you have it… (Fonhapo-LI)

In the Fonhapo FGD, there was not only a participant with a dengue diagnosis but also some of the participants belonged to a neighborhood health committee, and they had received training and information about this and other diseases. Both of these factors could have led to increased awareness about dengue transmission as compared to the other FGDs. The information people have about forms of transmission and spread of dengue are confusing, building a diffuse field of representation, even among those who have suffered from dengue directly. This forms an attitude toward the disease that may lead to stigmatization of people (through the isolation).

### Prevention strategies

In general, cleanliness was one of the primary factors in dengue prevention that was mentioned by the FGDs in all neighborhoods with either high- or low-dengue incidence. For example:

Cleaning our houses, yards, get Abate (Temephos) to use on the plants, the ones in pots, especially. (Insurgentes-HI).

The relationship between cleanliness and disease has a deep-rooted history, first introduced as the miasma theory that relates lack of cleanliness with certain diseases (15). In the case of dengue, the information shared by participants about the importance of cleanliness in prevention reiterates the need to maintain clean household spaces. Social messaging from the government may also shape this perception of prevention. *Patio Limpio* is one of the primary government sponsored campaigns to reduce dengue in which community members are encouraged to keep a clean yard to prevent the creation of mosquito habitat (16). Differential risk is perceived for people who undertake or do not undertake these measures.

However, these preventive measures are not localized only at the household level. Cleanliness of the community environment was also considered important. But at the community level, there are limits to the residents’ ability to undertake preventive actions. In all of the FGDs, the informants recalled situations of conflict with neighbors for not maintaining the cleanliness of their household space. Because of this, they recommended that the authorities impose a fine to coerce residents to keep household spaces clean*: For them to put some pressure on us, you’ll see that with a good fine* (Y Griega-LI).

Despite individual efforts, participants recounted that their prevention actions do not have corresponding support at the institutional level; and there is the perception that actions to reduce mosquito densities are limited, as mentioned in the Altares FGD:

We do a lot in the house so that the mosquito doesn’t settle, so that it doesn’t nest… but the government doesn’t support it, because if it supported it, it would say, let’s fumigate two or three times a year or prevent by giving Abate (Altares-LI).

At an institutional level, lack of preventive strategies has also been reported by health professionals in other regions of Mexico. These health professionals make reference to the importance of coordination among the community, health personnel, and the authorities (17). Maintaining cleanliness is, on one hand, viewed as an individual family’s responsibility while conducting fumigation and Abate supply campaigns are recognized as prevention strategies that should be undertaken by authorities. Abate is a larvicide, Temephos, that can be applied to water storage containers including water used for drinking to kill the immature stage of the mosquito vector of dengue. Beyond these concrete forms of dengue prevention, there are others that are tied to structural conditions that place distinct social groups at varying degrees of risk based on their socio-economic conditions. For participants from Minitas and Insurgentes neighborhoods, both with historically high dengue occurrence, access to services such as piped water and drainage are central to their concepts of disease prevention.

Although it doesn’t depend so much on us, also the government, for example, they don’t fix leaks or lack of drainage. In my case there is no drainage in the house and people have a moat and they dispose of the washing machine and bathroom water in the street… (Minitas-HI)

Lack of piped water in some neighborhoods has complicated the control of dengue. Some sections in both the Mintas and Insurgentes neighborhoods have piped water and drainage in the households; however, other households that have been established for more than 20 years still need to store water and request water truck services. These communities reported the importance of cleaning water storage containers and using Abate.

Regarding this discrepancy between knowledge and actual practices, Pinto (18) documents finding potential mosquito breeding sites within households, even though individuals indicate that they execute measures to keep their premises clean. This does not suggest that blame be placed on individuals, but rather it exposes how knowledge of what they are supposed to do may influence their reporting and intentions of prevention practices.

In both high- and low-transmission areas, participants recount that government campaigns are generally implemented to *control* an outbreak, not to *prevent* one from occurring. They note:

It is very obvious when there is dengue in the neighborhood because we hear the fumigation truck afterwards. They only send it when there is a case of dengue, that’s when they send the fumigation truck.

*As soon as there is a case like that there is a guy dispensing abate (Fonhapo-LI)*. For the participants, these actions indicate a failure on the part of the public sector to proactively address and/or eradicate the problem. The results overall provide a voice to the people and their discussions can be used to help define community-level prevention strategies that simultaneously promote social participation.

## Discussion and Conclusion

According to the Social Representation theory, the ideas, beliefs, and meanings about a social fact are the action guidelines of the social groups’ practices. Participants were confused about the mode of dengue transmission. This was particularly true in communities where dengue cases clustered in space and time, giving the appearance of direct transmission. In these communities, there was the misconception that dengue can be transmitted directly from person to person.

This study revealed that community members perceived transmission to occur even through contact with inanimate objects, known as fomites. For example, practices undertaken when a person dies from dengue, such as throwing away the bed of the person and their clothes, are related to a social representation of dengue as a disease caused by a miasma. Additionally, the belief that dengue can be transmitted from person to person suggests individuals may be making an association between dengue and other infectious diseases, such as influenza with which they may have had a longer history and more experience. Thus, people incorporate information and beliefs from other diseases into their interpretation of the transmission of dengue (18).

Future information and/or education campaigns should emphasize that dengue can only be transmitted through the bite of an infected mosquito. The concept of isolating individuals from interacting with other people should be discouraged as they could lead to rejection or social isolation as one of the participants mentioned. Although this experience was not repeated in other FGDs, future investigations should inquire about the stigma related to the disease. These beliefs could influence the uptake of prevention measures and may lead to stigma of dengue as is evidenced in some of the discussions. Stigma of a disease can complicate prevention and control efforts, alter treatment seeking behavior, and openness of discussion about the disease (19, 20). Even if stigma toward dengue is uncommon, alternative modes of dengue transmission are also mentioned in the FGDs where there were participants who had had dengue or who had cared for people who had had dengue. This reveals a significant gap in the information being passed from doctors to patients and their caregivers during diagnosis. These missed opportunities may also lead to negative outcomes due to a lack of follow-up of treatment and effective means of self-care.

Participants’ perception that isolating individuals with dengue from mosquito bites would reduce further spread could provide a significant opportunity for furthering prevention. If a viremic individual sleeps underneath a bednet, this could theoretically reduce new dengue virus infections in local mosquito populations that might have come into contact with the viremic person. Recent evidence suggests bednets can effectively reduce dengue (21). This concept of isolation from mosquitoes as a preventive strategy arose spontaneously from FGD, suggesting that it might be an acceptable strategy for communities to employ if it were determined to be effective in this setting. It is clear that participants’ felt individual action was the realm which they controlled and this type of household level protective measure could be acceptable.

We also found themes centered on social action, or more specifically, on the need to provide social participation in the prevention of the disease. Because of this, we look to emphasize the importance of this w/i/c process as a necessary component for the design of dengue prevention and eradication strategies. Under a social participation focus and according to Caballero (22) cultural significance should guide prevention practices, beyond the guidelines and biomedical prescriptions. Because community members recognized the need for action beyond just their own households, actions that mobilize the community toward social participation, such as clean-up campaigns may be successful. Social participation often varies by community, and higher rates of participation are associated with more success in reducing *Ae. aegypti* habitat in other regions of the world where dengue is endemic (23, 24). Clean-up for dengue control should involve a community-based approach, since the efforts at the household level do not reduce *Aedes* breeding sites in nearby areas (25). Strategies implemented in Mexico, such as Patio Limpio should be accompanied by educational messages about the storage of water, especially in communities where access to piped water is scarce and inappropriate storage practices may increase dengue risk. Also, dengue prevention should be approached using an environmental health model, promoting the active participation of the community, while improving access to public services (26). In this paper, we emphasize the social viewpoint of dengue and how it affects populations that do not have public services and basic health care.

Dengue is an emerging threat in the U.S.-Mexico border region. Understanding the community-level perceptions and beliefs about the disease can assist in developing strategies to improve understanding and reduce risk. As evidenced in 2014 when the first cases of dengue occurred in Caborca, San Luis Rio Colorado, and Nogales, dengue transmission is not static. Expansion into neighboring communities with less experience with dengue is going to occur and social connections with residents in areas with regular seasonal transmission, such as Hermosillo, will play a role in shaping the perspectives of the newly affected communities. Ensuring consistent and accurate messaging and engaging communities should be a critical component of all dengue prevention and control campaigns.

## Conflict of Interest Statement

The authors declare that the research was conducted in the absence of any commercial or financial relationships that could be construed as a potential conflict of interest. The Guest Associate Editor Cecilia Ballesteros Rosales declares that, despite being affiliated to the same institution as authors Kacey C. Ernst and Pablo Reyes-Castro, the review process was handled objectively and no conflict of interest exists.

## Footnotes

^1^The abbreviations HI and LI will be used to refer high incidence and low incidence, respectively.
